# Nursing Homes: Affiliation to Large Chains, Quality and Public–Private Collaboration

**DOI:** 10.3390/healthcare10081431

**Published:** 2022-07-30

**Authors:** Inmaculada Aguiar-Díaz, María Victoria Ruiz-Mallorquí, Beatriz González-López Valcarcel

**Affiliations:** 1 Department of Financial Economy and Accounting, Faculty of Economy, Business and Tourism, University of Las Palmas de Gran Canaria, 35017 Las Palmas, Spain; inmaculada.aguiar@ulpgc.es; 2 Department of Quantitative Methods for Economics and Management, Faculty of Economy, Business and Tourism, University of Las Palmas de Gran Canaria, 35017 Las Palmas, Spain; beatriz.lopezvalcarcel@ulpgc.es

**Keywords:** nursing homes, quality, largest chains, commissioning

## Abstract

The objective of this paper was to estimate the influence of being affiliated with an NH chain on perceived consumer quality, and whether this relationship is affected by maintaining a collaboration agreement with public administrations. We used a combination of theoretical foundations: (1) From the consumer perspective, we focussed on online reviews of the quality of nursing homes (NHs); (2) from the industrial organisation literature, we proposed arguments regarding the advantages and disadvantages of belonging to a chain; (3) the theory of transaction costs was used to explain public–private collaboration. The study was carried out on a sample of 642 chain-affiliated Spanish NHs, with data from quality scores downloaded from the website topMayores.es. We distinguished between the six largest chains and the rest. We applied linear regression models. The results show that NHs affiliated with one of the largest NH chains obtained worse quality scores in the assessment made by users, although quality scores improved for the largest chains of NHs involved in an agreement with the public administration.

## 1. Introduction

The European Union forecasts that long-term care expenditure will keep pressing public finances until 2070 [1]. Nursing homes (henceforth NHs) are currently an essential component of the long-term care system, and one of the main concerns regarding NHs for consumers, managers, governments and researchers is their quality [2,3,4]. 

Having information available regarding quality is a key aspect that affects the market structure of consumer goods and services. However, due the existence of asymmetric information between suppliers and consumers, public reporting is very important. Many studies have analysed NH quality from a medical perspective [2], but the necessary assessment from the point of view of consumers (residents and families) is scarce. Online patient reviews (OPRs) are a good mechanism for capturing the consumer perspective of NH quality, and consumers use them in their selection process [5]. The previous research is focused on the USA, probably due to the existence of databases about NH quality [6,7,8,9,10]. However, outside of the USA, studies regarding NH quality are scarce. 

Related to their ownership structure, NHs can be private for-profit, private non-for-profit and public [11]. Since the 1990s, the use of competing private providers in the healthcare industry has been a common practice [12,13]. Moreover, in recent decades, there has been an increase in the growth of chain affiliation in the NH industry [11]. The affiliation of for-profit NHs with chains has been addressed in the literature that analyses the performance of NHs, especially in relation to financial performance [11,14,15,16] and, to a lesser extent, in relation to quality [12,17,18]. Belonging to a chain allows the standardisation of certain care processes, which can contribute to improving [19] or worsening the quality [17]. Furthermore, in the case of large chains, managers focus on the interests of the shareholders over the needs of the residents [13,20]. Therefore, we say that membership in a large chain reduces quality. 

In addition, in many developed countries, the public administration has the responsibility of delivering long-term healthcare funded totally or partially by public funds. The short supply of places in public NHs, together with the increase in demand derived from the increase in life expectancy, has led to the establishment of collaboration agreements between the public administration and private NHs [21,22]. Given the fact that supervision and controls may be more stringent for commissioned NHs due to public interest, one would expect to find higher quality in commissioned NHs.

Spain is a good lab for investigating the nursing home industry for several reasons. Firstly, the considerable increase in life expectancy has led to a significant increase in the 65 and over demographic within the population. In 2020, 21% of the Spanish population was 65 years or older, and this is set to increase to 26.5% of the total population by 2035 (INE) (INE is the Spanish acronym of Spanish Statistic Institute. Available at https://www.ine.es/dyngs/INEbase/es/operacion.htm?c=Estadistica_C&cid=1254736176953&menu=ultiDatos&idp=1254735572981 (accessed on 26 July 2022)). Secondly, in Spain, the 10 biggest chains of NHs by revenue manage 27% of places in the private sector, rising to 35% for the 20 biggest [23]. Thirdly, commissioning agreements represent a traditional form of public–private collaboration in the Spanish healthcare industry [24]. In 2020, in Spain, 36% of total private NHs were commissioned [25]. 

In this context, the present work proposes a double objective: first, to contribute towards reducing the existing gap in the literature regarding the study of the quality of NHs; second, to analyse the influence of large, affiliated chains on NHs in Spain, as well as the possible moderating effect of maintaining an agreement with the public administration on the relationship between belonging to a large chain and quality. The results obtained from a sample of 642 Spanish NHs reveal that NHs affiliated with one of the largest NH chains obtained worse quality scores in the assessment made by users, although quality scores improved for NHs that had an agreement with the public administration. 

## 2. Literature Review

### 2.1. NH Quality from the Consumer Perspective. Online Reviews

Information available about quality is a key aspect that affects the market structure of consumer goods and services. If information about quality is imperfect and asymmetric, the providers of goods and services can react by underproviding on quality [8]. Therefore, the existence of ways to reduce the information gap about the quality of good and services, such as public reporting or online reviews, seems crucial for providers (supply-side) in order to maintain or increase market share [8]. Additionally, for consumers (demand-side), public information about quality enables them to choose better-ranked providers [26].

Furthermore, the consumer perspective on healthcare is becoming more accepted as a valid measure of quality, complementing traditional clinical measures [27]. Specifically, in healthcare, the greater importance and popularity of online review platforms means that consumers can easily inform other potential consumers about their experiences so as to form a consumer-oriented perspective [5,28]. As a consequence, we focussed our analysis on consumer-determined quality.

The quality of NHs is multidimensional as they provide care in medical and social dimensions, among others. Thus, some NH quality indicators try to reflect different aspects (regarding the NHs´ quality, there are many studies made in the USA based on public reporting, specifically using the NHC ratings. In [8], a wide review of studies based on this public reporting is presented). These aggregated indicators may not be suitable for the measurement of the quality of care for each dimension due to the differences among them and, often, they have a medical focus without considering other valuable dimensions for consumers [2]. In this sense, consumer ratings of care would reflect a dimension of quality and interpersonal care experiences not well represented by clinical indicators [29].

According to this, residents’ and families’ satisfaction represents a way to consider the consumer voice as an indicator of quality. Specifically, families are more concerned about aspects such as cleanliness, the availability of activities, communication between nursing staff and families and the adequacy and respectfulness of staff, while medical care is less important [27,29]. Furthermore, regarding NHs, the role of families is more relevant because many residents are psychiatrically and/or cognitively impaired [29,30]. However, residents and relatives could be fearful of the reactions of the healthcare providers if they report negative evaluations—perhaps more relevant in the case of long-term stays [29].

Related to the consumer/patient quality perspective in the healthcare and social care areas, patients may use online patient reviews (OPRs), which represent an unsolicited, free and continuously updated, social media-based source of information about patient or family experience [5,29] and are associated with consumer satisfaction. The OPR platforms are easy to use and accessible. In fact, OPRs may reach more population groups that have been less represented in traditional surveys [5]. Typically, their structure contains some sort of rating and open text to describe consumers’ experiences and support their selected rating.

Among the studies that used an online platform to analyse the quality of NHs are [31,32,33]—all of them are cross-sectional studies, referring to the USA. The first uses Yelp, the second Facebook and the third uses the two social networks, Google Review and Caring.com (a review of the studies that used online patient reviews can be found in [5]).

### 2.2. Chain-Affiliated NH and NH Quality 

A for-profit NH is chain-affiliated if it is a member of a multifacility organisation [13,14]. Specifically, there is a chain of NHs when a firm owns or manages two or more NHs [12,18]. According to the theory of industrial organisation, the main reasons to explain the rise in chains in a competitive environment would be [11,12,15,17,34]: the economies of production that may result in lower input costs and the more efficient use of resources; the economies of finance that allow increased access to capital; the economies of promotion that may reduce the consumer’s search for price and quality information; and an increase in their market power. Along the same line, Banaszak-Holl et al. [17] argue that “chains may transfer skills as R & D capabilities, production know-how, use of specific technologies, applications of new products, marketing skills, management of supplier and regulatory relations, management of distribution channels, and administrative procedures” (263). The growth in chain affiliation in the NH industry in recent decades is a sign of evolution towards more structured and corporatised arrangements [11].

The affiliation of for-profit NHs with chains has been addressed in the literature that analyses the performance of NHs, especially in relation to financial performance [11,14,15,16] and, to a lesser extent, in relation to quality [12,17,18]. 

Regarding the relationship between NHs’ affiliation with a chain and their quality, on the one hand, the growth of NH chains in the long-term care industry has led to concern that operating cost reductions through standardisation may reduce the quality of service offered by NHs [17,20]. The main reason to explain this negative relationship would be that profit seeking diverts funds and focus away from clinical care [20]. On the other hand, Grabowski et al. [19] assert that the same standardisation of care practices may improve quality. In this sense, Banaszak-Holl et al. [17] argue that the discipline and experience of corporate managers and the availability of new operating routines in target homes could explain the major benefits of chain affiliation.

The existence of controversial ideas regarding the effect of chain affiliation in quality may be a consequence of considering all chain-affiliated NHs in a single category encompassing all sizes and structures [35]. Some studies have focused on NHs’ affiliation to the largest chains. In this vein, most of those studies found a negative relationship between affiliation with the largest chains and quality [12,13,18]. Those results are possibly due to the fact that those large chains focus on corporate interests over residents’ needs and use strategies to maximise shareholder and investor returns at the expense of resident care [13,18]. Furthermore, large chains, thanks to their ability and resources for developing active marketing campaigns, can attract consumers regardless of their quality [18]. Finally, large chains might be less concerned about sanctions for poor quality because they have more resources to fight against them and view those sanctions as a normal cost of business [13].

Hence, based on the previous arguments, we argue that NHs affiliated with the largest NH chains have lower levels of quality than NHs affiliated with other chains:

**H1.**  *Affiliation with the largest NH chains reduces the quality of the NH.*

However, it is possible that some of the negative aspects derived from affiliation with a large chain can be partially compensated when the NH maintains collaboration agreements with the public sector, since those agreements include a number of requirements, including maintaining a certain standard of quality. Through these agreements, private healthcare providers receive payments from public budgets for providing services to publicly insured patients. For example, in the USA, the Medicaid and Medicare programmes pay for about 75% of all NHs’ residents [36]. In Spain, although there are differences among the autonomous communities, on average, 70% of the price of a commissioned place is provided by the public administration [37].

Regarding the requirements, for example in the USA, the law requires the provision of the same level of quality to all residents regardless of the payer source (Medicaid or private) [38,39]. Similarly, in Spain, all NHs must achieve some standards of service, defined by central and regional regulations on several aspects (material resources and equipment, staff, documentation and information).

Given that supervision and controls may be more stringent for commissioned NHs due to public interest, one would expect higher quality in commissioned NHs. Along this line, Bowblis [40] analyses the higher levels of requirement placed on NHs by public bodies regarding quality and concludes that NHs with Medicaid have fewer deficiencies.

In addition to the legal aspects, and from the economic point of view, the theory of transaction costs can explain public–private collaboration. Following this theory, the contracts between the public and private sectors try to reduce the information asymmetries, and their effectiveness will depend on the relationship between the obtained benefits and the costs associated with the definition and proper supervision of those contracts [41]. For the private healthcare sector, this commissioning may increase potential demand, contributing to the improved performance of healthcare providers, as long as they are able to contain costs and align public tariffs for commissioned services [24]. Regarding quality, [38] concludes that there is a positive relationship between reimbursement and quality in most NHs in the USA. 

Therefore, NHs with a commissioning agreement in particular will be forced to focus more on the needs of residents, as well as to be more concerned with quality in order to attract new residents, even if they have sufficient resources to withstand penalties for poor quality. This can help to reduce the negative effect of their affiliation with a large chain. Consequently, we argue that NHs affiliated with the largest chains that have a commissioning agreement would present better quality than the non-commissioned ones:

**H2.**  *Commissioning improves the relationship between the largest NH chains and quality.*

## 3. Research Method

### 3.1. Sample and Sources of Information

The sample was extracted from the online database topMAYORES.es, which contains a list of 105 groups, as well as the NHs affiliated with each of them. For each NH, it offers a star rating based on the opinions of users or their relatives, as well as the location of the NH with the address, the number of places and the commissioning status. Some of the NHs managed by groups are public, although the vast majority are private. The initial sample of NHs linked to groups is *n* = 1013, of which 92 are public, reducing the sample to 921 private NHs. Of these, 279 do not have a quality score, so the final sample is made up of 642 private NHs managed by 90 groups. The source of perceived quality is the website topMAYORES.es, an online search platform for NHs. Other sources used are the INE (for provincial GDP per capita) and the Web of Aging in network (https://envejecimientoenred.es/ (accessed on 15 February 2022)) (for the number of NHs by province).

### 3.2. Variables

Dependent variable: Quality. NH quality is proxied by the average score given by users, which in a broad sense includes residents or their families [8]. The averaged opinions may have been posted at any time, unlike regular surveys, so the number and date of evaluations made by users differ among NHs. The platform uses five-star quality ratings (one to five stars), and more stars indicate better quality. The data used correspond to the average score of the ratings posted by the different users. 

#### Explanatory Variables: Big6 and Commissioning

Largest NH chain affiliation (Big6) is a dichotomous variable = 1 if the NH is affiliated with one of the six largest NH private for-profit Spanish chains, and 0 if the NH is affiliated with another chain (according to the literature on NH, both those owned and managed by the group are considered chain affiliates [12,18]). The selection of these groups was made based on the ranking of the total number of places managed by the different chains [12,18], as well as by the volume of billing (data provided by Valverde, 2021). Likewise, six dummies were created, one for each of the large chains (Amavir, Ballesol, DomusVi, Orpea, Sanitas y Vitalia) (the allocation criterion used consisted of assigning to each chain all the NHs that include the name of the chain in their name, regardless of the name of the management company. For example, the NHs included in DomusVi are managed by DomusVi, Gerovida, Geriatros, SARquavitae and Sarrikue. Those of Orpea are Orpea and Ecoplar). These six chains jointly manage some 500 NHs in Spain, with more than 56,000 beds, and had a turnover of more than EUR 1300 million in 2021 [21]. Foreign capital predominates, specifically French (DomusVi controlled 100% by HomeVi. S.A.S.; Amavir, with 81.81% ownership in the hands of Groupe Maisons de Famille; Orpea, a subsidiary of Orpea, S.A. and Sanitas, wholly owned by BUPA; Vitalia, controlled 80% by CVC Capital Partners). The only group controlled by Spanish capital is Ballesol (Compañía de Seguros Santa Lucía, which holds 75% ownership).

Commissioning (COM) is a dichotomous variable that takes the value 1 if the NH maintains a commissioning agreement for which a public administration (Social Services) is responsible for paying for all or part of the services provided by the NH on an individual basis, i.e., for at least some of the places in the NH. In order to analyse the moderating effect of management by a Big6 chain with the existence of an agreement with public services, the variable Big6xCOM was created, which takes the value 1 if the NH is managed by a Big6 and it also has a commissioning agreement. Likewise, 5 interaction variables were created between each of the Big6 and the agreement dummy (DomusvixCOM, BallesolxCOM, OrpeaxCOM, VitalixCOM and SanitasxCOM), except Amavir, since all the NHs belonging to that group have all their places under agreement.

Control variables. According to the literature on NH quality (e.g., [6,15,18,30]), the size should be considered as a control variable. We proxy it with the logarithm of the number of places. Size is an important structural factor that influences the performance of organisations, generating benefits derived from economies of scale and the greater capacity to obtain resources [15]. Other variables to control for are market factors, namely the socioeconomic level, the degree of concentration and the potential public. As an indicator of socioeconomic conditions, we used the logarithm of GDP per capita of the province in which the NH is located (data from 2019) [6,15]. Competition at the provincial level was approximated by the Herfindahl–Hirschman Index (HHI), calculated from the number of existing places (public and private) in each province (year 2020). It is the sum of the squares of the market shares (in %) of all NHs in the province. HHI values range between 0 and 10,000 points, and the lower the index is, the more competitive the market becomes.

Finally, regarding the estimation method, we applied a linear regression model, specifically Ordinary Least Squares (OLS), since the dependent variable (quality) can take continuous values as it is an average of the scores given by the users.

## 4. Results

### 4.1. Descriptive Analysis

The percentage of NHs managed by a Big6 is around 60% of the sample. Of those, 75% have an agreement with the public administration, ranging from 11% (Vitalia) to 100% (Amavir). Only 47% of NHs managed by a non-Big6 chain have a commissioning agreement. Globally, 64% of NHs have a commissioning agreement with the public administration (Table 1).

In terms of geographical location, a large concentration is observed in a few provinces. In particular, Madrid occupies the first position with 24%, followed by Barcelona with 13%. As can be seen in Table 1, certain differences are observed in the presence and provincial concentration of the chains. Analysing the sum of the three provinces with the highest number of residences, there is a higher average concentration of the No Big6 than the Big6 NHs. Moreover, between the largest chains, the highest concentration occurs in Amavir, Orpea and Sanitas with more of 70% of their NHs in three provinces.

Table 2 presents the data related to the quality of the residencies. The mean score given to the residencies is 3.26 points out of a maximum of 5, being higher for the NHs with an agreement (3.29) than for the rest (3.19), although this difference is not statistically significant. In addition, differences are observed between the NHs managed by one of the Big6 (mean score 3.16) and the rest (3.39), with this difference being statistically significant. Among the NHs managed by a Big6, there is a significant difference in the perceived quality in favour of the ones with a commissioning agreement (3.27 vs. 2.83).

The differences between NHs with and without agreements are only statistically significant for the subgroup of NHs managed by a Big6. In addition, there are significant differences between specific Big6 chains. Amavir achieves greater satisfaction, followed by Domusvi and Ballesol, while Orpea has the lowest score. It should be noted that Amavir, the chain with the highest score, and the only one that exceeds the average of NHs managed by smaller chains, has all its NHs under agreement. Two Big6 NH chains (Domusvi and Vitalia) perform significantly better under agreement.

Table 3 presents the correlation matrix and the VIF. The average size of the NHs is 134 places (logarithm = 4.78). This figure is slightly higher in the Big6 and for NHs under agreement. The average provincial GDP per capita is around EUR 28,200 (logarithm = 10.22). The average provincial HHI is 118.70 median = 88, (range: 21–568), which indicates a low level of concentration. As can be seen, there are no high correlations between the explanatory variables, and, consequently, there should be no serious multicollinearity problems.

### 4.2. Econometric Results

In order to test the hypotheses proposed, we estimated models 1 and 2, considering the entire sample and controlling for the size of the NH, provincial GDP per capita and HHI. Table 4 presents the results. Model 1 includes the Big6 dummy, while in models 2 and 3, this variable is replaced by the six dummies corresponding to each of the large chains, so that the reference category is an NH managed by a non-Big6 group. According to model 1, being managed by one of the large chains (Big6) reduces perceived quality (0.41 points below an NH managed by a smaller chain, *p*-value < 0.01). Likewise, having an agreement only improves quality significantly when the NH is managed by a Big6, with an increase of 0.46 points (*p*-value < 0.05).

In model 2, the Big6 dummy is replaced by the six specific dummies, and there is no interaction between the latter and commissioning. Commissioning is positive and significant at 10%. Three of the six specific chain dummies are significant: Orpea and Vitalia are negative and Amavir is positive. Model 3 adds the interactions between commissioning and the specific dummies of the Big6 to model 2. The results reveal some differences between the chains. Only Amavir (100% of its NHs under commission) has a perceived quality higher than the reference NH (No Big6 chain). The remaining five have a negative sign, although it is only statistically significant for three of them: Domusvi and Vitalia (at 1%), and Orpea (at 10%). Commissioning is not significant for the whole sample, but it is positive and significant for two of the six big chains (Domusvi and Vitalia). Regarding the control variables, in all the models, the largest NHs located in provinces with greater concentration and greater GDP per capita have poorer quality, with different levels of significance.

To analyse the robustness of the results obtained, model 1 was re-estimated for the subsamples of NHs managed by a Big6 (model 4) and not managed by a Big6 (model 5) (See Table 5). Commissioning is statistically significant and only positive in the subsample of NHs managed by a Big6. Lastly, we estimated models 6 and 7 for the subsamples of NHs with and without commissioning. 

The results maintain the sign and significance of the Big6 for the subsample of NHs without commissioning. For the subsample of NHs with commissioning, Big6 is positive but not significant.

Finally, we also analysed the robustness of the results considering the possible selection bias derived from the elimination of NHs without a quality score. Specifically, all the models 1–3 were re-estimated using the Heckman method [42] for correcting selection bias. Results are available under request. All results are very close to those in Table 4, and they do not show selection bias, as the estimate of the inverse Mills ratio obtained in the first stage probit model is not significant.

## 5. Discussion

In this study, we examined the quality scores of NHs belonging to chains in Spain. Users and customers posted those evaluations on a webpage over time. We investigated two non-exclusive factors that could influence perceived quality: belonging to one of the six largest NH chains (Big6) and having a commissioning agreement with the public administration to provide services to residents who are entitled to public coverage under the Law 39/2006. Both theoretical considerations, along with some previous empirical studies for other countries, helped formulate our two hypotheses: (H1) affiliation with the largest NH chains reduces NH quality; (H2) commissioning improves the relationship between the largest NH chains and quality.

The results obtained offer support to H1 when we introduce the dummy Big6 as an explanatory factor in the regression model. In this sense, our results are consistent with Harrington et al. [18] in the USA and also the findings of Harrington et al. [12] in several other countries (Canada, Sweden, the UK and the USA), and You et al. [35] in the USA. However, when we consider each of these large chains individually, this effect is only relevant in two of the six considered. In addition, the case of Amavir stands out as an exception, as it has the highest average score, even exceeding the average of NHs belonging to smaller chains. In addition, this chain is characterised by having all its centres under commission, only being present in the eight provinces with the highest concentration—82% of their NHs are located in three provinces, one of these being Madrid—and 81% being controlled by the French Groupe Maisons de Famille. Therefore, we can partially accept H1, and we conclude that Big6 chains are heterogeneous.

Regarding the moderating effect of commissioning, the results reveal that those NHs managed by a Big6 that have a commissioning agreement provide better quality than those without. Furthermore, this effect is more relevant for two of the Big6, DomusVi and Vitalia, both controlled by foreign capital—French and British, respectively. Therefore, hypothesis 2 is partially accepted. 

## 6. Conclusions and Practical Implications 

Given that the presence of large NH chains is an increasing trend in developed countries, its potential negative impact on quality has become a big concern [17]. Therefore, the analysis of NH quality is a key factor to be considered by the managers of these NHs, by their users and by the public authorities that reach commissioning agreements with them. 

Our results show that—in Spain, at least—not all large chains present low levels of quality (driven by the pressure towards profits and increases in company value). Therefore, the managers of these NH chains should make an effort to convey to users and their families that they are not neglecting quality. At the same time, managers should bear in mind that commissioning agreements may attract more users to NHs, but they also require higher levels of quality.

On the other hand, due to the growing presence on the market of large NH chains, public authorities should pay special attention to them in order to avoid distortions in terms of quality. Our results show that the existence of a commissioning agreement improves quality; also, for this reason, public authorities should not neglect resources for the inspection and control of commissioned NHs and increase sanctions where necessary.

According to the study carried out by Domínguez et al. [43] over the period 2014–2019, the nursing home system in Spain is characterised as an opaque system, incurring numerous small fines for infractions against which there is very little recourse for users; this is a system with inspections that are insufficient in their regularity, which leaves users practically defenceless in the face of the often frivolous penalties that are frequently imposed. The aforementioned study indicates that 25% of the sanctions are for a lack of personnel and 75% are for serious or very serious offences. In addition, the fines are in the low range of what is legally permitted. In most communities, the most common fine is EUR 5000.

## Figures and Tables

**Table 1:** Distribution of the sample of private NHs affiliated to a chain.

	Initial Sample	Final Sample	NHs with Agreement (%)	Number of Provinces	Provincial Concentration (% of Top 3 NHs)
Total	921	642	63.86	49	45.02
No Big6	423	260	47.31	39	50.77
Big6	498	382	75.13	46	42.15
DomusVi	273	173	17.34	32	31.79
Ballesol	41	39	65.62	16	48.72
Amavir	35	34	100.00	8	82.35
Orpea	63	60	28.33	17	65.00
Vitalia	40	36	11.11	14	47.22
Sanitas	46	40	57.50	14	70.00

**Table 2:** Descriptives.

	All		With Commissioning (COM)		No Commissioning (NoCOM)		*t*-Test
	Average	SD	Average	SD	Average	SD	COM_NoCOM
Total	3.26	1.14	3.29	1.13	3.19	1.17	−1.16
No Big6	3.39	1.21	3.35	1.21	3.43	1.20	0.54
Big6	3.16	1.09	3.27	1.09	2.83	1.03	−3.44 ***
*t*-test Big6 vs. no Big6	2.49 **		0.63		3.93 ***		
DomusVi	3.33	1.12	3.47	1.04	2.64	1.20	−3.87 ***
Ballesol	3.15	0.86	3.12	1.10	3.17	0.60	0.15
Amavir	3.92	0.81	3.92	0.81	-	-	-
Orpea	2.50	0.92	2.37	0.84	2.83	1.07	1.75 *
Vitalia	2.85	1.05	2.97	1.03	1.87	0.63	−2.06 **
Sanitas	3.09	0.17	3.28	1.18	2.95	1.04	−0.95
K-Wallis	47.24 ***		49.12 ***		9.19 *		11.52 ***

K-Wallis test of equality of the six Big chains. *, ** and ***: significant at 10%, 5% and 1%, respectively.

**Table 3:** Correlations and Variance Inflation Factor (VIF).

	Score	Big6	Commis.	Size	GDP p.c.	HHI
Average	3.2578	0.5950	0.6386	4.7828	10.2225	118.7048
SD	1.1457	0.4912	0.4807	0.5587	0.2304	100.7908
VIF		1.24	1.10	1.14	1.42	1.44
Score	1.0000					
Big6	−0.0983 *	1.0000				
Commissioning	0.0458	0.2843 ***	1.0000			
Size (log)	−0.1868 ***	0.3453	0.1563 ***	1.0000		
GDPper capita (log)	−0.0180	−0.0753 *	0.0256	0.0221	1.0000	
HHI (log)	−0.0610	0.1444 ***	0.0657 *	−0.0012	−0.5394 ***	1.0000

K-Wallis test of equality of the six Big chains. *, *** significant at 10% and 1%, respectively.

**Table 4:** Econometric results.

	Model 1		Model 2		Model 3	
	β	S.E.	β	S.E.	β	S.E.
Big6	−0.4153 ***	−0.1555	-	-	-	-
Commissioning	−0.0099	0.1395	0.1745 *	0.0979	−0.0136	0.1348
Big6xCOM	0.4642 **	0.1922	-	-	-	-
DomusVi	-	-	0.0154	0.1184	−0.5644 **	0.2254
Ballesol	-	-	−0.0833	0.1975	−0.0932	0.2585
Amavir	-	-	0.6925 ***	0.2102	0.7698 ***	0.2138
Orpea	-	-	−0.7479 ***	0.1623	−0.4638 *	0.2814
Vitalia	-	-	−0.4371 **	0.2032	−1.4153 ***	0.5497
Sanitas	-	-	−0.1642	0.1876	−0.3621	0.2449
DomusVixCOM	-	-	-	-	0.7717 ***	0.2568
BallesolxCOM	-	-	-	-	−0.0099	0.3723
OrpeaxCOM	-	-	-	-	−0.3532	0.3399
VitaliaxCOM	-	-	-	-	1.1755 **	0.5880
SanitasxCOM	-	-	-	-	0.4148	0.3704
Size	−0.3564 ***	0.0846	−0.3468 ***	0.0829	−0.3184 ***	0.0827
HHI	−0.0011 **	0.0005	−0.0014 ***	0.0005	−0.0014 ***	0.0005
GDP per capita	−0.3892 *	0.2280	−0.4991 **	0.2299	−0.4446 *	0.2296
Constant	9.1222 ***	2.3864	10.1506 ***	2.4010	9.5435 ***	2.4012
N. Obs	642		642		642	
R-squared	0.0599		0.1128		0.1347	

Dependent variable: quality score. Estimation method: OLS linear regression. *, ** and ***: significant at 10%, 5% and 1%, respectively.

**Table 5:** Econometric results. Robustness.

	Model 4		Model 5		Model 6		Model 7	
Subsample	Big6		No Big6		With Commissioning		No Commissioning	
	β	S.E.	β	S.E.	β	S.E.	β	S.E.
Big6	-	-	-	-	0.1009	0.1252	−0.5028 ***	0.1648
Commissioning	0.4558 ***	0.1259	−0.0254	0.1506	-	-	-	-
Size	−0.4424 ***	0.1242	−0.3066 **	0.1202	−0.5111 ***	0.1146	−0.1802	0.1296
HHI	−0.0015 **	0.0006	−0.0004	0.0010	−0.0012 **	0.0006	−0.0013	0.0009
GDP per capita	−0.4336	0.2773	−0.3009	0.3918	−0.2952	0.2732	−0.5231	0.4142
Constant	9.6306 ***	2.9136	7.9276 *	4.0906	8.8779 ***	2.8536	9.7227 **	4.3458
N. Obs	382		260		410		232	
R-squared	0.0732		0.0286		0.0535		0.0805	

Dependent variable: quality score. Estimation method: OLS linear regression. *, ** and *** significant at 10%, 5% and 1%, respectively.

## Data Availability

Not applicable.
